# Application of preventive medicine resources in the health insurance system

**DOI:** 10.1590/S1679-45082015GS3453

**Published:** 2015

**Authors:** Karla Regina Dias de Oliveira, Márcia Mello Costa De Liberal, Paola Zucchi

**Affiliations:** 1 Universidade Federal de São Paulo, São Paulo, SP, Brazil.

**Keywords:** Preventive medicine, Supplemental health, Insurance, health, Prepaid health plans, Financial resources in health, Investments

## Abstract

**Objective:**

To identify the financial resources and investments provided for preventive medicine programs by health insurance companies of all kinds.

**Methods:**

Data were collected from 30 large health insurance companies, with over 100 thousand individuals recorded, and registered at the *Agência Nacional de Saúde Suplementar*.

**Results:**

It was possible to identify the percentage of participants of the programs in relation to the total number of beneficiaries of the health insurance companies, the prevention and promotion actions held in preventive medicine programs, the inclusion criteria for the programs, as well as the evaluation of human resources and organizational structure of the preventive medicine programs.

**Conclusion:**

Most of the respondents (46.7%) invested more than US$ 50,000.00 in preventive medicine program, while 26.7% invested more than US$ 500,000.00. The remaining, about 20%, invested less than US$ 50,000.00, and 3.3% did not report the value applied.

## INTRODUCTION

With increased longevity one notes an increase in concerns regarding creating initiatives, so that people might enjoy health and quality of life in all phases of life. Within this context, we aimed to study the effect of the preventive medicine programs in healthcare provided by health insurance companies in Brazil.

According to data from the Ministry of Health,^(1)^ more than 85% of elderly Brazilians presented with at least one chronic disease, whereas approximately 15% of this age range recorded at least five of them. Additionally, the elderly 60 aged years or more consume, in terms of hospitalization, between two and four times as much resources than young and adult patients. Thus, the great challenge the healthcare system faces for the next decades will be to care for a contingent estimated at approximately 32 million elderly individuals, with a high prevalence of chronic and disabling diseases, and a high financial and social cost.^(2)^

In the current context, the growth of the elderly population has made evident the lack of preparation of the healthcare system to deal with the peculiarities of these individuals. The first aspect that stands out is the fact that healthcare assistance is organized according to a model that privileges treatment of acute cases and episodes, such as those that affect the younger population.^(3)^ Considering the variation of costs, the health insurers, attentive to the demographic changes occurring, to the profile of their clients, and to the guidelines of the *Agência Nacional de Saúde Suplementar *(ANS), are implementing health prevention and promotion programs. Nevertheless, to date, we have not observed studies in the literature describing the characteristics and costs resulting from these operations and procedures.

This aspect was what instigated the interest in this research to contribute towards knowledge of information on health prevention and promotion programs, offering new parameters for studies on feasibility and costs of these initiatives. This effort to study the actions focused on private health insurance plans in Brazil is fundamental, since the National Public Health System (SUS – *Sistema Único de Saúde*) demands increasingly more financial resources, required by a society more mature in age and in the capacity to demand it.^(4)^

Sestelo et al.^(5)^ prepared a systematic review on private health insurance plans in Brazil, based on the normative/temporal mark of law 9,656/98 and the creation of ANS, which reviewed the studies published on this theme between 2000 and 2010, leading to the identification of 4,700 titles. This expressive number in reference to the performance of research in the most diverse academic levels reveals the concern with contributing so that this research field might occupy an increasingly more important analytical space that stands out in the Brazilian social scenario.

Within this context, we highlight that health insurance or private healthcare segment, as it is also called, is responsible for a large portion of the population; however, the expenses with treatment of diseases in their acute phase demonstrates that, from an economical point of view, if there is no investment in prevention and promotion of health in the country, this system will also fail.

The scenario described above has led the ANS to orient the healthcare insurers to adopt measures capable of collaborating for the prevention and promotion of this sector, which leads to the questioning that this study aimed to answer, considering the evaluation and analysis of the effect of the healthcare plans, in terms of investment in preventive medicine programs.

## OBJECTIVE

To identify the financial resources and the investments available for the preventive medicine programs in health insurers of all types.

## METHODS

The field research was carried out with the health insurers registered at the ANS, which take part and are evaluated by the Private Health Plan Qualification Program. The material investigated was made up of the health insurers registered with the ANS. Considering this, we understand the importance of reporting these data.

This is a cross-sectional study that included health insurers with more than 100 thousand beneficiaries, providing care nationwide at hospitals, and classified the Private Health Plan Performance Index, which was evaluated in 2008. Data were collected through a semi-structured questionnaire^(6)^ with 13 closed questions on the health prevention programs developed by the insurers operators. The questions addressed the aspects related to resources involved and characteristics of the program. The questionnaire is attached to the indicated dissertation and was electronically forwarded to the selected insurers by e-mail. They e-mailed the filled out questionnaires. Along with the questionnaire, the Informed Consent Form was sent, to comply with resolution 196/96, to offer to those investigated information about the study, including respect and confidentiality in use of data. The study was submitted to the Ethics Committee of *Universidade Federal de São Paulo* (UNIFESP), and was approved under number 0584/09, of May 15, 2009.

## RESULTS

A total of 71 questionnaires were sent to the insurers, and only 42.2% answered and returned them. Of these, 60% opted for not enrolling their preventive medicine programs with the ANS (Table 1).

**Table 1:** Enrolment of preventive medicine programs at the *Agência Nacional de Saúde Suplementar* in 2008

Enrolment at the ANS	n (%)
Yes	12 (40)
No	18 (60)

Total	30 (100)

ANS: *Agência Nacional de Saúde Suplementar.*

As shown in table 2, when analyzing the participation of the beneficiaries in the preventive medicine programs of the insurers, 86.7% of the programs had no participation of all beneficiaries.

**Table 2:** Insurers with participation of the total number of beneficiaries in the preventive medicine program

Insurers with participation of all beneficiaries	Quantity n (%)
Yes	4 (13.3)
No	26 (86.7)

Total	30 (100)

Only 13.3% of the insurers investigated informed that all their beneficiaries participated in the preventive medicine program.

The most predominant diseases among the participants of the preventive medicine programs were *diabetes mellitus* (16.1%), hypertension (15.6%), dyslipidemia (14%), other heart diseases (10.2%), stroke (9.7%), myocardial infarction (9.1%), chronic obstructive pulmonary disease (8.60%), cancer (7%), osteoporosis (4.3%), and osteoarthritis (2.7%). In the category “others diseases” (2.7%), obesity/overweight, asthma, pregnancy, smoking habits, functional spinal lesions, and mood disorders were reported. The age ranges that benefited from the preventive medicine programs varied from very young to the eldest, as shown on table 3.

**Table 3:** Age groups involved in preventive medicine programs

Age group (years)	Quantity n (%)
0-19	12 (7.6)
20-29	22 (14)
30-39	23 (14.7)
40-49	25 (15.9)
50-59	26 (16.6)
60-69	25 (15.9)
70 or more	24 (15.3)

Total	157 (100)

When the health insurers were asked about participation in preventive medicine programs, the main criterion adopted for including the beneficiary was types of diseases (21.3%), followed by presence of risk factors (20.5%), analysis of cost-benefit (19.7%), contract losses (17.3%), age (15%), and sex (6.3%).

The investment made by the insurers for the program varied, in 2008, by up to over US$ 500,000.00 among the companies investigated, as displayed on table 4.

**Table 4:** Investments in the preventive medicine program

Value (US$)	Quantity of insurers n (%)
10,000.00 - 15,000.00	1 (3.3)
20,001.00 - 25,000.00	1 (3.3)
25,001.00 - 35,000.00	2 (6.7)
35,001.00 - 50,000.00	2 (6.7)
Over 50,001.00	14 (46.7)
Over 500,000.00	8 (26.7)
Other	1 (3.3)
Not informed	1 (3.3)

Total	30 (100)

Most health insurers invested over US$ 50,000.00 in the preventive medicine program in 2008, and 26.7% of them invested more than half a million dollars, while 20% invested less than US$ 50,000.00 and only one did not inform the value invested.

As to operationalization of the preventive medicine program, more than half, that is, 54.5% of them used their own resources, whereas 27.3% divided this task with the contractors and 18.2% outsourced.

Most private insurance companies evaluated the results of preventive medicine programs, and the method most often mentioned was control of hospital admissions (21.9%), followed by cost reduction (21.1%), control in the use of the healthcare plan (16.7%), evaluation of quality of life by means of application of questionnaires (16.7%), variation in portfolio losses (10.5%), goals proposed and reached (8.8%), and others (2.6%). It is worth mentioning that 2.6% of those investigated did not provide this information.

The absence of details regarding the distribution of resources and quantity of beneficiaries hinders an evaluation as to the sufficiency of the investment. Some insurers privileged the elderly who were already in a chronic situation, aiming to control worsening of the condition and the occurrence of acute events. Among the programs used by the companies, those most frequent were lectures, followed by telephone contact, campaigns with explanatory leaflets, home visits, besides follow-up of the patient’s profile, as well as vaccination.

## DISCUSSION

According to the ANS, during the period of the study, there were 1,042 private health insurance companies under operation. One important fact to point out is the change in the incentive of preventive medicine programs in a timeline. During the decade of the 1970s, only one health insurer had a preventive medicine program under operation. Almost two decades later, three private health insurance companies implemented these programs. Nevertheless, it was only in 2002 that more than 85% of the operators reported establishing preventive medicine programs, which reveals a structural behavioral change regarding health-related prevention, which contributes to administrative cost reduction and certainly provides more control and quality to healthcare services.^(7)^

The problems that the health insurers face regarding preventive medicine programs are related to the fact that these companies were not used adequately prepared for their structuring, modeling, and management.^(6)^ Additionally, there are difficulties relative to geographic dispersion and compliance of the beneficiaries. The ANS, in agreement with the policies of the Ministry of Health, guides the development of programs in different care areas, such as child, adolescent, young adult, adult, worker, woman, man, and the elderly health, among others.^(8)^

In the same way, monitoring and evaluation of the programs are fundamental for their optimized management and learning of new interventions.^(9)^ The majority of the programs take into consideration the elderly population, which is consistent with the increased consumption of services by these individuals. This aspect becomes even more expressive when added to the increased life expectancy, which leads to understanding that the chronic and degenerative conditions will worsen over time. Due to these characteristics and to diversity of factors influencing quality of life in each region of the country, this study has possible limitations that should be considered in the general evaluation of the results.

## CONCLUSION

Most of the health insurance companies assess results of preventive medicine programs, and the most often used methods are control of hospital admissions, cost reduction, control of use of the health insurance plan, evaluation of quality of life by applying questionnaires, variation in portfolio losses, and the establishment of the goals proposed and reached.

The registration of the preventive medicine programs at the *Agência Nacional de Saúde Suplementar *is not unanimous and compliance of the users with this plan is low.

The diseases included for participation in the programs were diabetes mellitus, hypertension, dyslipidemia, other cardiac diseases, stroke, myocardial infarction, chronic obstructive pulmonary disease, cancer, osteoporosis and osteoarthritis. In the category “other diseases”, obesity/overweight, asthma, pregnancy, smoking habits, functional spinal lesions, and mood disorders were mentioned.

The investment for conducting the program varied by up to over US$ 500,000.00 among the companies investigated. All health insurers have a professional responsible for coordinating/management of the preventive medicine programs, and most of them are physicians and nurses.
