# Supplementary material for: Application of preventive medicine resources in the health insurance system
Source: Einstein (Sao Paulo). 2015 Oct-Dec;13(4):600–3. doi: 10.1590/S1679-45082015GS3453 (PMC4878637; doi:10.1590/S1679-45082015GS3453)
Supplement: Translation

# Translation: Aplicação de recursos em medicina preventiva no sistema de saúde complementar

**Authors:** Karla Regina Dias de Oliveira, Márcia Mello Costa De Liberal, Paola Zucchi

**Affiliations:** 1 Universidade Federal de São Paulo, São Paulo, SP, Brasil.

**Keywords:** Medicina preventiva, Saúde suplementar, Seguro saúde, Planos de pré-pagamento em saúde, Recursos financeiros em saúde, Investimentos em saúde

## Abstract

**Objetivo:**

Identificar os recursos financeiros e os investimentos disponibilizados para os programas de medicina preventiva em operadoras de saúde suplementar de todos os tipos.

**Métodos:**

Foram levantados dados referentes a 30 operadoras de saúde registradas na Agência Nacional de Saúde Suplementar, de grande porte, com registro acima de 100 mil vidas.

**Resultados:**

Foi possível identificar o porcentual de participantes dos programas em relação ao número total de beneficiários da operadora, as ações de prevenção e promoção realizadas nos programas de medicina preventiva, os critérios de inclusão nos programas, bem como a avaliação dos recursos humanos e da estrutura organizacional dos programas de medicina preventiva pesquisadas.

**Conclusão:**

A maior parte dos pesquisados (46,7%) investiu mais de US$ 50,000.00 no programa de medicina preventiva, enquanto 26,7% investiram mais de US$ 500,000.00. Os restantes, cerca de 20%, investiram menos de US$ 50,000.00 e 3,3% não informaram o valor aplicado.

## INTRODUÇÃO

Com o aumento da longevidade, observa-se um crescimento das preocupações em criar iniciativas para que as pessoas possam desfrutar de saúde e qualidade de vida em todas as fases da vida. Nesse contexto, procurou-se estudar a atuação dos programas de medicina preventiva na saúde suplementar no Brasil.

Segundo dados do Ministério da Saúde,^(1)^ mais de 85% dos idosos brasileiros apresentam pelo menos uma doença crônica, enquanto aproximadamente 15% dessa faixa etária registra até cinco delas. Além disso, os idosos com 60 anos ou mais consomem, em internações, entre duas a quatro vezes mais recursos do que os pacientes jovens e adultos. Assim, o grande desafio com que se depara o sistema de saúde nas próximas décadas é cuidar de um contingente estipulado de cerca de 32 milhões de idosos com alta prevalência de doenças crônicas, incapacitantes e um elevado custo financeiro e social.^(2)^

Na atual conjuntura, o crescimento da população idosa tem evidenciado o despreparo do sistema de saúde, para lidar com as peculiaridades desses indivíduos. O primeiro aspecto que sobressai é o fato de a assistência à saúde ser organizada em um modelo que privilegia o tratamento de casos agudos e episódicos, como os que acometem a população mais jovem.^(3)^ Em função das variações dos custos, as operadoras de planos de saúde, atentas às mudanças demográficas em curso, ao perfil de sua carteira de clientes e às orientações da Agência Nacional de Saúde Suplementar (ANS) estão implementando programas de prevenção e promoção da saúde. No entanto, até o momento, não se observaram na literatura estudos que divulguem as características e os custos decorrentes dessas operações e procedimentos.

Esse aspecto foi o que despertou o interesse desta pesquisa, ao possibilitar contribuir para o conhecimento das informações sobre os programas de prevenção e promoção da saúde, oferecendo novos parâmetros para estudos de viabilidade e do custo dessas iniciativas de programas. Esse esforço em estudar as ações focadas na saúde suplementar, por meio das operadoras de saúde no Brasil, é fundamental, uma vez que o Sistema Único de Saúde (SUS) demanda, cada vez mais, recursos financeiros exigidos por uma sociedade mais madura na idade e na capacidade reivindicativa.^(4)^

Sestelo et al.^(5)^ elaboraram uma revisão sistemática sobre saúde suplementar no Brasil, a partir do marco normativo/temporal da lei 9.656/98 e da criação da ANS, que revisou os estudos publicados sobre esse tema, entre 2000 e 2010, permitindo identificar 4.700 títulos. Esse número expressivo, no que se refere à realização de pesquisas nos mais diversos níveis acadêmicos, revela a preocupação de contribuir para que essa área de pesquisa ocupe um espaço analítico cada vez mais importante e merecedor de destaque no cenário social brasileiro.

Neste contexto, cabe destacar aqui que o segmento de saúde suplementar ou privada, como também é denominado, é responsável por uma grande parcela da população; porém, os gastos com tratamento de doenças em seu estágio agudo demonstram que, do ponto de vista econômico, se não houver investimento na prevenção e na promoção da saúde no país, esse sistema também vai sucumbir.

O cenário aqui relatado tem levado a ANS a orientar os planos de saúde a adotarem medidas capazes de colaborar para a prevenção e a promoção desse setor, o que conduz ao questionamento que este estudo buscou responder, diante da avaliação e da análise sobre a atuação das operadoras de planos de saúde, em termos de investimento em programas de medicina preventiva.

## OBJETIVO

Identificar os recursos financeiros e os investimentos disponibilizados para os programas de medicina preventiva em operadoras de saúde suplementar de todos os tipos.

## MÉTODOS

A pesquisa de campo foi realizada junto às operadoras registradas na ANS, participantes e avaliadas pelo Programa de Qualificação da Saúde Suplementar. O material de análise pesquisado foi constituído pelas operadoras que tinham registro na ANS de números de dados. Isso posto, entendemos a importância da divulgação destes dados.

Trata-se de um estudo transversal que incluiu operadoras com mais de 100 mil beneficiários; com operação nacional; prestação de assistência médico-hospitalar e classificação no Índice de Desempenho da Saúde Suplementar, realizado em 2008. Para tanto, foi elaborado um instrumento para a coleta de dados, na forma de um questionário^(6)^ semiestruturado com 13 questões fechadas, sobre os programas desenvolvidos pelas operadoras selecionadas para a prevenção em saúde, que abordou aspectos relacionados aos recursos envolvidos e características do programa. Ele encontra-se inserido na dissertação indicada e foi encaminhado às operadoras selecionadas por meio eletrônico (*e-mail*), sendo essa também a forma de devolução após o preenchimento. Juntamente do questionário, foi encaminhado um Termo de Consentimento Livre e Esclarecido, visando, além do atendimento à resolução 196/96, oferecer aos pesquisados informações a respeito da pesquisa e do respeito e sigilo na utilização das informações obtidas. O estudo foi encaminhado e recebeu parecer favorável do Comitê de Ética da Universidade Federal de São Paulo (UNIFESP) número 0584/09, de 15 de maio de 2009.

## RESULTADOS

No total, foram enviados questionários para 71 operadoras, sendo que somente 42,2% deles retornaram respondidos. Destas, 60% das operadoras optaram por não inscrever seus programas de medicina preventiva na ANS (Tabela 1).

**Tabela 1:** Inscrição dos Programas de Medicina Preventiva na Agência Nacional de Saúde Suplementar em 2008

Inscrição na ANS	Quantidade n (%)
Sim	12 (40)
Não	18 (60)

Total	30 (100)

ANS: Agência Nacional de Saúde Suplementar.

Conforme a tabela 2, quando se analisou a participação dos beneficiários nos programas de medicina preventiva das operadoras pesquisadas, 86,7% dos programas não tinham participação de todos os beneficiários.

**Tabela 2:** Operadoras com participação do total de beneficiários no programa de medicina preventiva

Operadoras com participação de todos os beneficiários	Quantidade n (%)
Sim	4 (13,3)
Não	26 (86,7)

Total	30 (100)

Somente 13,3% das operadoras pesquisadas informaram que todos os beneficiários participavam do programa de medicina preventiva.

Na análise das doenças mais predominantes entre os participantes dos programas de medicina preventiva, aparecem o *diabetes mellitus* (16,1%), a hipertensão arterial (15,6%), a dislipidemia (14%), outras doenças do coração (10,2%), acidente cerebrovascular (9,7%), antecedente de infarto do miocárdio (9,1%), doença pulmonar obstrutiva crônica (8,60%), câncer (7%), osteoporose (4,3%) e osteoartrose (2,7%). Na categoria “outras doenças” (2,7%), foram citados: obesidade/sobrepeso, asma, gestação, tabagismo, lesões funcionais da coluna e distúrbios psíquicos do humor. As faixas etárias beneficiadas pelos programas de medicina preventiva variaram desde as muito jovens até as mais idosas, como mostra a tabela 3.

**Tabela 3:** Faixas etárias contempladas pelos programas de medicina preventiva

Faixa etária (anos)	Quantidade n (%)
0-19	12 (7,6)
20-29	22 (14)
30-39	23 (14,7)
40-49	25 (15,9)
50-59	26 (16,6)
60-69	25 (15,9)
70 ou mais	24 (15,3)

Total	157 (100)

Quando as operadoras foram questionadas sobre a participação em programas de medicina preventiva, o principal critério adotado para inclusão do beneficiário foi aquele que considera os tipos de doenças (21,3%), seguido da presença de fatores de risco (20,5%), da análise do custo do benefício (19,7%), da sinistralidade dos contratos (17,3%), da idade (15%) e do sexo dos beneficiários (6,3%).

O investimento realizado pelas operadoras para a realização do programa variou em até mais de US$ 500,000.00 entre as empresas pesquisadas, como pode ser observado na tabela 4, no decorrer do ano de 2008.

**Tabela 4:** Investimento realizado no programa de medicina preventiva

Valor (US$)	Quantidade de operadoras n (%)
10,000.00 - 15,000.00	1 (3,3)
20,001.00 - 25,000.00	1 (3,3)
25,001.00 - 35,000.00	2 (6,7)
35,001.00 - 50,000.00	2 (6,7)
Mais de 50,001.00	14 (46,7)
Mais de 500,000.00	8 (26,7)
Outro	1 (3,3)
Não informou	1 (3,3)

Total	30 (100)

A maior parte das operadoras investiu mais de US$ 50,000.00 no programa de medicina preventiva em 2008 e 26,7% delas investiram mais de meio milhão de dólares, enquanto 20% investiram menos de US$ 50,000.00 e somente uma não informou o valor investido.

Quanto à operacionalização do programa de medicina preventiva, mais da metade, ou seja, 54,5% delas utilizaram recursos próprios enquanto 27,3% dividiram essa tarefa com os contratados e 18,2% contrataram terceiros.

A maior parte das operadoras realizava avaliações dos resultados dos programas de medicina preventiva, sendo que o método mais citado foi o de controle de internações (21,9%), seguido de redução de custos (21,1%), controle na utilização do plano de saúde (16,7%), avaliação da qualidade de vida por meio da aplicação de questionários (16,7%), variação na sinistralidade da carteira (10,5%), metas propostas e alcançadas (8,8%) e outros (2,6%). Ressalta-se que 2,6% dos pesquisados não prestaram essas informações.

A ausência de detalhes a respeito da distribuição dos recursos e da quantidade de beneficiários contemplados dificultou uma avaliação sobre a suficiência do investimento. Algumas operadoras privilegiaram os idosos que já se encontravam em situação crônica, visando controlar o agravo das doenças e a ocorrência de quadros agudos. Entre os programas utilizados pelas empresas, destacaram-se as palestras, seguidas do contato telefônico, das campanhas com folhetos explicativos, da visita domiciliar, além do acompanhamento do perfil do paciente, assim como da prática da vacinação.

## DISCUSSÃO

De acordo com a ANS, existiam, no período do estudo, 1.042 operadoras de planos de saúde médico-hospitalares em atividade. Um fato importante a ser destacado é a mudança no incentivo aos programas de medicina preventiva na linha do tempo. Na década de 1970, apenas uma operadora de plano de saúde mantinha programa de medicina preventiva em operação. Quase duas décadas depois, três operadoras implementaram esses programas. No entanto, foi somente a partir de 2002 que mais de 85% das operadoras informaram a criação de programas de medicina preventiva, o que revela uma mudança estrutural de comportamento em relação à preocupação com a saúde em seu aspecto preventivo, contribuindo para redução de custos administrativos e trazendo, certamente, mais racionalidade e qualidade para a prestação do serviços de saúde.^(7)^

Os problemas que os programas de medicina preventiva enfrentam nas próprias operadoras estão relacionados ao fato de essas empresas não estarem instrumentalizadas de forma adequada para sua estruturação, sua modelagem e seu gerenciamento.^(6)^ Além disso, são encontradas dificuldades relativas à dispersão geográfica e à adesão dos beneficiários. A ANS, em consonância com as políticas do Ministério da Saúde, orienta o desenvolvimento de programas em diferentes áreas de atenção à saúde, como, por exemplo, a saúde da criança, do adolescente, do jovem, do adulto, do trabalhador, da mulher, do homem e do idoso, entre outras.^(8)^

De igual modo, o monitoramento e a avaliação dos programas são fundamentais, para que seja otimizada sua gestão e que ocorra aprendizado de novas intervenções.^(9)^ A maior parte dos programas contempla a população idosa, o que é consistente com o aumento do consumo de serviços e recursos apresentados por esses indivíduos. Esse aspecto torna-se mais expressivo quando se adiciona o aumento da expectativa de vida, que induz ao entendimento de que as patologias de curso crônico e degenerativas devem se acentuar com o passar dos anos. Em virtude dessas características e da diversidade dos fatores que influenciam na qualidade de vida em cada região do país, este trabalho apresenta possíveis limitações que devem ser consideradas na avaliação geral dos resultados.

## CONCLUSÃO

A maior parte das operadoras de saúde realiza avaliações dos resultados dos programas de medicina preventiva, sendo os métodos mais utilizados o controle de internações, a redução de custos, o controle na utilização do plano de saúde, a avaliação da qualidade de vida por meio da aplicação de questionários, a variação na sinistralidade da carteira e o estabelecimento das metas propostas e alcançadas.

O cadastramento dos programas de Medicina Preventiva na Agência Nacional de Saúde Suplementar não é unânime, e a adesão dos usuários a esse plano é baixa.

As doenças contempladas para a participação nos programas foram diabetes mellitus, hipertensão arterial, dislipidemia, outras doenças do coração, derrame ou acidente cerebrovascular, antecedente de infarto do miocárdio, doença pulmonar obstrutiva crônica, câncer, osteoporose e osteoartrose. Na categoria “outras doenças”, foram citadas obesidade/sobrepeso, asma, gestação, tabagismo, lesões funcionais da coluna e distúrbios psíquicos do humor.

O investimento para a realização do programa variou em até mais de US$ 500,000.00 entre as empresas pesquisadas. Todas as operadoras pesquisadas contavam com um profissional responsável pela coordenação/gerenciamento dos programas de medicina preventiva, e a maioria era de médicos e enfermeiros.
